# Prevalence, Virulence Feature, Antibiotic Resistance and MLST Typing of *Bacillus cereus* Isolated From Retail Aquatic Products in China

**DOI:** 10.3389/fmicb.2020.01513

**Published:** 2020-07-03

**Authors:** Ying Zhang, Mengfei Chen, Pengfei Yu, Shubo Yu, Juan Wang, Hui Guo, Junhui Zhang, Huan Zhou, Moutong Chen, Haiyan Zeng, Shi Wu, Rui Pang, Qinghua Ye, Liang Xue, Shuhong Zhang, Ying Li, Jumei Zhang, Qingping Wu, Yu Ding

**Affiliations:** ^1^Department of Food Science and Technology, Institute of Food Safety and Nutrition, Jinan University, Guangzhou, China; ^2^State Key Laboratory of Applied Microbiology Southern China, Guangdong Provincial Key Laboratory of Microbial Safety and Health, Guangdong Open Laboratory of Applied Microbiology, Guangdong Institute of Microbiology, Guangzhou, China; ^3^College of Food Science, South China Agricultural University, Guangzhou, China

**Keywords:** aquatic products, *Bacillus cereus*, prevalence, MLST, multi-drug resistance

## Abstract

*Bacillus cereus* is one of the most important foodborne pathogenic microorganisms, which can lead to gastrointestinal and non-gastrointestinal diseases. However, the potential risk of *B. cereus* in aquatic products in China has not been comprehensively evaluated yet. In this study, a total of 860 aquatic samples from three types of retail aquatic products were collected from 39 major cities in China from 2011 to 2016. The contamination, distribution of virulence genes, antibiotic resistance and genetic diversity of *B. cereus* isolates were measured and analyzed. Of all the samples, 219 (25.47%) were positive for *B. cereus* and 1.83% (4/219) of the samples had contamination levels of more than 1,100 most probable number (MPN)/g. Different isolates had virulence potential, within which 59.6% (164/275) contained all three kinds of enterotoxin genes (*nhe*, *hbl*, and *cytK-2*) and 5.1% (14/275) possessed cereulide encoding gene *cesB*. The antimicrobial resistance profiles revealed the universal antibiotic resistance to rifampin and most β-lactams, suggesting the necessity to continuously monitor the antibiotic resistance of *B. cereus* in aquatic products and to control drug use in aquaculture. In sum, our study indicates the potential hazards of *B. cereus* isolated from aquatic products to customers and may provide a reference for clinical treatment caused by *B. cereus*.

## Introduction

As one of the key sources of nutrition, aquatic products are an indispensable part of consumers’ diets (Thilsted et al., 2014). But, the problem of bacterial contamination in aquatic products always exists (Kim et al., 2017). Consuming raw aquatic products like sashimi is now very popular around the world, making foodborne diseases caused by bacterial contamination in aquatic products more likely (Miguéis et al., 2015). *Bacillus cereus*, a facultative aerobic and spore-forming Gram-positive bacterium, is a well-known foodborne opportunistic pathogen (Bottone, 2010; Messelhäußer and Ehling-Schulz, 2018). Since *B. cereus* and its dormant spores are widely present in nature (Andersson et al., 1995; Bottone, 2010), it can easily contaminate different types of food. When *B. cereus* exceeds 10^5^ CFU/g, it is considered to be unacceptable/potentially hazardous (Gilbert et al., 2000). The contamination incidents of *B. cereus* in aquatic products have been reported previously (Rahmati and Labbé, 2008; Iwamoto et al., 2010; Miguéis et al., 2016).

As *B. cereus* in aquatic products may cause food poisoning, it is necessary to investigate the prevalence and potential hazards of different isolates. Food poisoning symptoms induced by *B. cereus* include diarrhea and vomiting (Stenfors Arnesen et al., 2008), which are mainly caused by non-hemolytic enterotoxin (Nhe), hemolysin BL (Hbl), cytotoxin K (CytK) (Stenfors Arnesen et al., 2008; Messelhäußer and Ehling-Schulz, 2018), and a cyclic dodecadepsipeptide named cereulide (Agata et al., 1995; Ehling-Schulz et al., 2015). The symptoms of gastrointestinal infections caused by *B. cereus* are generally acute and mild. However, *B*. *cereus* can also lead to some severe non-gastrointestinal infections, such as endophthalmitis, bacteremia, septicemia, meningitis, and pneumonia (Hoffmaster et al., 2006; Bottone, 2010; Rishi et al., 2013; Veysseyre et al., 2015). Currently, the most common treatment for the infections and severe food poisoning by *B. cereus* is antibiotic therapy. If the strain is resistant to the antibiotics used clinically, it will cause the failure of the treatment. Thereby, the resistance profiles of *B. cereus* isolates to different antibiotics could be used as a reference for the clinical curing.

As the risk of *B. cereus* in aquatic products in China has not been comprehensively evaluated yet, we aimed to analyze the prevalence of *B. cereus* in this study, as well as the molecular characteristics (virulence genes, antibiotic resistance profiles, and genetic diversity) of different isolates to explore the potential hazard of *B. cereus* in aquatic products in major cities of China.

## Materials and Methods

### Sampling

From 2011 to 2016, 860 aquatic products were collected from one supermarket and two traditional retail markets in 39 major cities (Supplementary Figure S1) according to the Chinese general guidelines of food microbiological examination (The Hygiene Ministry of China, 2010). After collection, aquatic samples were placed in sterile plastic bags, immediately transported back to the laboratory at low temperature (below 4°C) and subjected to further test and analysis. Microbial experiments were operated in class II biosafety cabinets in a BSL2 laboratory.

### Isolation and Identification of *B. cereus*

Qualitative and quantitative detection of *B. cereus* were performed according to the food microbiological examination guidelines of *B. cereus* (The Hygiene Ministry of China, 2003) and previous studies (Gao et al., 2018; Yu et al., 2019, 2020) with minor modification. Briefly, 25 grams of aquatic sample were homogenized at 8,000 to 10,000 rpm about 2 min in a sterile bag (Huankai, Guangzhou, China) with 225 mL 0.01 mol/L phosphate-buffered saline (PBS). The homogenates were incubated for 48 ± 2 h at 30 ± 2°C. Then the cultures were streaked onto mannitol-egg yolk-polymyxin (MYP) agar plates and incubated for 24 h at 30°C. Single colonies were then streaked onto chromogenic*B. cereus* agar plates (Huankai, Guangzhou, China). Typical colonies were further confirmed with biochemical testing (Gao et al., 2018; Yu et al., 2019, 2020). *B. cereus* ATCC 14579 was used as a reference strain for biochemical characterization. The most probable number (MPN) method was used for the quantitative detection of *B. cereus.* The detailed procedures were performed as previously described (Yu et al., 2020) and the MPN table is listed in Supplementary Table S1 (The Hygiene Ministry of China, 2014).

ERIC-PCR was used to characterize the clonal isolates of *B. cereus* identified from the same sample (Gao et al., 2018; Yu et al., 2020). The primer set (named ERIC-F and ERIC-R; Versalovic et al., 1991; Gao et al., 2018) is listed in Supplementary Table S2. If two or more isolates from the same sample had the exact fingerprint, only one of the strains was kept for further test and the others were excluded as clonal isolates.

### Detection of Virulence Genes

Seven enterotoxigenic genes (*hblA*, *hblC*, *hblD*, *nheA*, *nheB*, *nheC*, and *cytK-2*) and one emetic toxin-producing gene (*cesB*) were detected by PCR using the primers listed in Supplementary Table S2 (Hansen and Hendriksen, 2001; Ehling-Schulz et al., 2005; Oltuszak-Walczak and Walczak, 2013). Genomic DNA was extracted using a HiPure Bacterial DNA extraction kit (Magen, Guangzhou, China) under the instruction of the manufacturer. Concentration and purity of the DNA were measured by Nanodrop 2000 spectrophotometer (Thermo Fisher Scientific, United States). The amplification reactions were performed using ExTaq Mix kit (Takara, China) in a Biometra TOne 96G thermal cycler (Analytik Jena, Jena, Germany). The PCR reaction mixture (25 μL) contained 50 ng of genomic DNA, 1.0 μM of each primer and 12.5 μL ExTaq Mix. Amplification was performed according to the instruction of the manufacturer (ExTaq Mix, Takara, China).

### Testing of Antibiotics Susceptibility

Antimicrobial susceptibility of different strains was evaluated by the Kirby-Bauer (KB) disk diffusion method, which was performed and interpreted as described by the Clinical and Laboratory Standards Institute (The Clinical and Laboratory Standards Institute [CLSI], 2010) and previous publications (Gao et al., 2018; Yu et al., 2019). Twenty antibiotics (Oxoid, United Kingdom; Supplementary Table S3) were selected based on the performance standards for antimicrobial susceptibility testing of the CLSI for *Staphylococcus aureus* (The Clinical and Laboratory Standards Institute [CLSI], 2010). The diameter of the inhibition zone (Supplementary Table S3) was measured to evaluate the antibiotic resistance of different isolates.

### Discrimination of Psychrotrophic and Mesophilic Strains

Rapid discrimination of psychrotrophic and mesophilic *B. cereus* was done by the detection of the 16S rDNA signatures (von Stetten et al., 1998; Stenfors and Granum, 2001). The primers were listed in Supplementary Table S2 and the PCR program was conducted as described previously (von Stetten et al., 1998) with modifications. The amplification was performed using DreamTaq Green PCR Master Mix (2X) (Thermo Fisher Scientific, United States) following the instruction of the manufacturer.

Growth test of 16 strains, including 14 *cesB*-positive isolates from aquatic products, *B. cereus* ATCC14579 and a clinical emetic-type strain *B. cereus* F4810/72, was conducted according to the previous publication (Luu-Thi et al., 2014) with minor modifications. A single colony of each isolate on BHI agar was inoculated into a 5 mL of BHI broth and then incubated at 30°C until the OD_600_ reaching 0.6–0.8 (logarithmic growth phase). Afterward, 100 μL of bacterial culture were sprayed on BHI agar plates and incubated at either 7or 43°C within 20 days. Three biological replicates of each strain were performed. If visible colonies could form on the plate incubated at 7°C, the strain was considered to be psychrotrophic isolate.

### Multilocus Sequence Typing (MLST) and Phylogenetic Analysis

Seven housekeeping genes were amplified with corresponding primers and conditions as described by the protocol available in PubMLST^1^. In general, the PCR amplification system (25 μL), referring to the instruction manual of PrimeSTAR Max Premix (Takara, China), contained 12.5 μL PrimeSTAR Max Premix, 50 ng genomic DNA, 1.0 μM of each primer. The sequence type (ST) of each isolate was obtained as described previously (Yu et al., 2019, 2020). A minimal spanning tree was created using PHYLOViZ 2.0 software (Instituto de Microbiologia, Portugal; Nascimento et al., 2017) to visualize the possible evolutionary relationships between different isolates for epidemiological analysis based on MLST alleles. Phylogenetic analysis between the sequence types (STs) of 275 isolates and eight type strains (*B. cereus* ATCC 14579, *Bacillus mycoides* DSM 2048, *Bacillus pseudomycoides* DSM 12442, *Bacillus weihenstephanensis* WSBC 10204, *Bacillus anthracis* ATCC 4728, *Bacillus thuringiensis* ATCC 10792, and two clinical emetic-type strains *B. cereus* NC7401 and *B. cereus* F4810/72) was conducted using the BioNumerics software (version 7.6; Applied Maths, Belgium) by the unweighted pair group method of arithmetic averages (UPGMA) method with a 52% similarity level.

## Results

### Prevalence Analysis of *B. cereus* in Aquatic Products

The prevalence and contamination level of *B. cereus* in 860 samples are shown in Table 1. The aquatic products we collected can be divided into three categories: (i) finfish; (ii) mollusks; and (iii) crustaceans (Iwamoto et al., 2010). Through the ERIC-PCR method, 13 strains from 288 were excluded as they present clonal strains (Supplementary Figure S2). Overall, 275 *B. cereus* isolates were detected in 25.47% (219/860) of the samples, with 25.62% in finfish, 23.32% in mollusks, and 27.78% in crustaceans. In terms of collecting sites, the contamination rate in 15 cities was ≥ 30.00% and even reached 50.00% in Shaoguan (Supplementary Table S4). About the contamination level, 79.45% (174/219) of the samples ranged from 3 to 1,100 MPN/g. In 1.83% (4/219; all from finfish) of total samples, the contamination level exceeded 1,100 MPN/g, indicating that *B. cereus* in these samples may have a higher risk to cause disease.

**TABLE 1:** Prevalence and contamination level of *B. cereus* isolated from aquatic products.

Type	Prevalence rate (%)^a^	MPN value (MPN/g)^b^		
		MPN < 3 (%)	3 ≤ MPN < 1100 (%)	1100 ≤ MPN (%)
Finfish	134/523 (25.62)	28/134 (20.90)	102/134 (76.12)	4/134 (2.99)
Mollusks	45/193 (23.32)	10/45 (22.22)	35/45 (77.78)	0/45 (0.00)
Crustaceans	40/144 (27.78)	3/40 (7.50)	37/40 (92.50)	0/40 (0.00)
Total	219/860 (25.47)	41/219 (18.72)	174/219 (79.45)	4/219 (1.83)

*^*a*^Prevalence rate = number of positive samples/total samples. ^*b*^MPN value (MPN/g) = most probable number of *B. cereus* per gram sample.*

### Distribution of Virulence Genes and Psychrotolerant Ability of Emetic Strains

**FIGURE 1:**
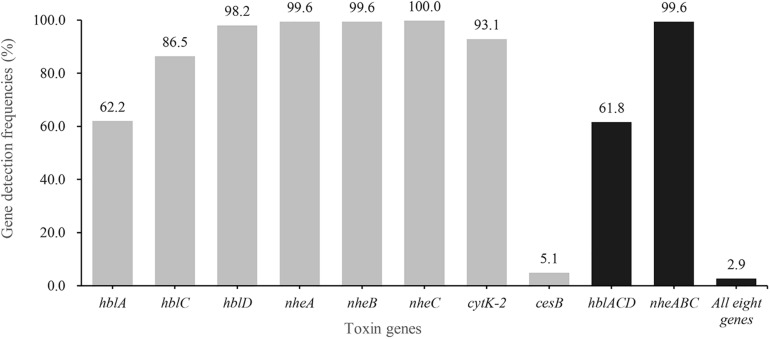
Detection rate of virulence genes in *B. cereus* from aquatic products. The gray bars of different heights represent the positive rate of *hblA, hblC, hblD, nheA, nheB, nheC, cytK*-2 and *cesB* in *B. cereus* isolates, respectively. The dark bars indicate the positive rate of *hbl* gene cluster (*hblA*, *hblC*, and *hblD*), or *nhe* gene group (*nheA*, *nheB*, and *nheC*), or all eight virulence genes.

The distribution of virulence genes is shown in Figure 1. More than 99.6% of the isolates harbored *nhe* genes and 61.8% of the isolates possessed *hbl* ones. Besides, *cytK*-*2* was present in 93.1% of the isolates. In contrast, only 14 isolates (5.1%) possessed cereulide synthetase gene *cesB*. Eight strains, named 875, 2039-1, 2078, 2931-1A, 3626-1B, 3729-2C, 3831-1A and 3927-1C, harbored all eight virulence genes (*nheA-nheB-nheC-hblA-hblC-hblD-cytK-2-cesB*; Supplementary Figure S3). About 60.0% (164/275) of the strains were found to contain genes encoding all three types of enterotoxins (*nhe*, *hbl* and *cytK-2*), which suggests that *B. cereus* strains isolated in this study have a higher potential to cause diarrheal disease.

The psychrotolerant ability of all *cesB*-positive strains was tested by amplification of specific signatures within 16S rDNA or by growth test at different temperatures. Although all strains showed specific bands representing mesophilic and psychrotrophic signatures within 16S rDNA (Table 2 and Supplementary Figure S4), all *cesB*-positive strains could grow at 43°C instead of 7°C, revealing their non-psychrotolerant identity (Guinebretière et al., 2008).

**TABLE 2:** Characteristics of *cesB*-positive isolates.

Strain	Growth at 7°C	Growth at 43°C	M	P	Source	Region
875	−	+	+	+	Finfish	E
2039-1	−	+	+	+	Finfish	E
2078	−	+	+	+	Crustaceans	N
2827-2A	−	+	+	+	Finfish	C
2829-1A	−	+	+	+	Crustaceans	C
2829-2A	−	+	+	+	Finfish	C
2931-1A	−	+	+	+	Finfish	SW
3626-1B	−	+	+	+	Finfish	SW
3629-1B	−	+	+	+	Mollusks	SW
3631	−	+	+	+	Finfish	SW
3726-3A	−	+	+	+	Finfish	E
3729-2C	−	+	+	+	Mollusks	E
3831-1A	−	+	+	+	Finfish	NE
3927-1C	−	+	+	+	Finfish	C
ATCC14579	−	+	+	+	−	−
F4810/72	−	+	+	+	−	−

*C, central China; E, east China; M, mesophilic specific signature; N, north China; NE, northeast China; NW, northwest China; P, psychrotrophic specific signature; S, south China; SW, southwest China.*

### Antimicrobial Susceptibility of *B. cereus* Isolates

**FIGURE 2:**
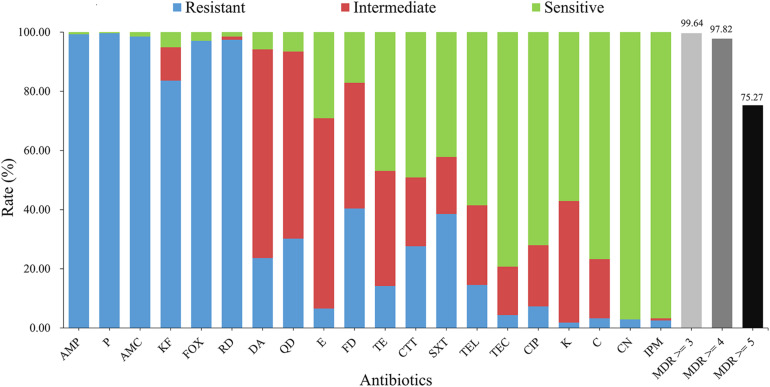
Antimicrobial characteristics of *B. cereus* from aquatic products. The blue, red, and green bars represent the proportion of resistant, intermediate resistant, and sensitive strains, respectively. The light gray, gray, and black bars show the proportion of strains with multidrug resistance (MDR) to at least three, four, and five classes of antibiotics, respectively. AMP, ampicillin (10 μg); P, penicillin (10 units); AMC, amoxicillin-clavulanic acid (20 μg/10 μg); KF, cephalothin (30 μg); FOX, cefoxitin (30 μg); RD, rifampin (5 μg); DA, clindamycin (2 μg); QD, quinupristin-dalfopristin (15 μg); E, erythromycin (15 μg); FD, nitrofurantoin (300 μg); TE, tetracycline (30 μg); CTT, cefotetan (30 μg); SXT, trimethoprim-sulfamethoxazole (1.25 μg/23.75 μg); TEL, telithromycin (15 μg); TEC, teicoplanin (30 μg); CIP, ciprofloxacin (5 μg); K, kanamycin (30 μg); C, chloramphenicol (30 μg); CN, gentamicin (10 μg); IPM, imipenem (10 μg).

According to the results of antimicrobial susceptibility test (Figure 2), nearly all isolates showed resistance to rifampin (RD; 97.5%) and most β-lactams [ampicillin (AMP; 99.3%), penicillin (P; 99.6%), amoxicillin-clavulanic acid (AMC; 98.5%), cephalothin (KF; 83.6%), and cefoxitin (FOX; 97.1%)], whilst they gave less resistance to other β-lactams, such as cefotetan (CTT) and imipenem (IPM) (27.6% and 2.5%, respectively). IPM (96.7%), gentamicin (CN; 97.1%), teicoplanin (TEC; 79.3%) and ciprofloxacin (CIP; 72.0%) could effectively inhibit the growth of different strains.

Regarding 133 antimicrobial resistance profiles (Supplementary Figure S3), 58 isolates (21.1%) were resistant to ≥10 antibiotics. The strain 1581-3C, 1705-1C and 1977-3 turned out to be the most highly resistant isolates, which were resistant to 13 antibiotics (Supplementary Figure S3). In contrary, the strain 3004-3A was only resistant to two antibiotics (FOX-RD). Further antimicrobial resistance profile analysis revealed that the most common one was AMP-P-AMC-KF-FOX-RD. Based on the definition of multi-drug resistance (MDR; Magiorakos et al., 2012), a very high proportion (99.64%) of isolates were MDR and 75.27% of the population were resistant to five types of antibiotics.

### Analysis of Genetic Diversity

**FIGURE 3:**
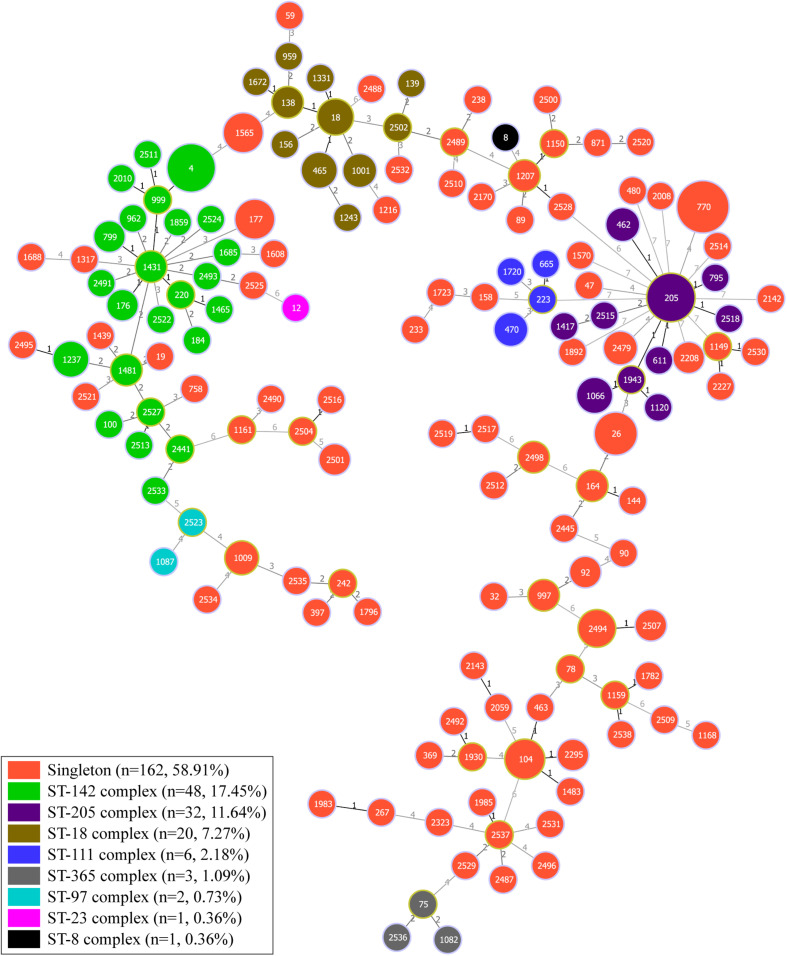
Minimum spanning tree and genetic diversity of 275 *B. cereus* isolates from aquatic products. Each color represents one clonal complex or a group of singletons. The numbers inside the pie chart are the corresponding sequence types (STs), and the size of the pie chart is proportional to the number of isolates in the corresponding ST. The number along the line indicates the variation of the seven loci between two strains at both ends of the line.

The minimal spanning tree was generated based on the sequences of seven housekeeping genes to estimate the relationships between different strains (Figure 3). Overall, 275 isolates were assigned with 147 STs, which contained 45 new STs (including 52 isolates) (Supplementary Table S5). ST770, the most predominant ST, included 28 isolates, followed by ST4 and ST205 with 18 isolates each. Furthermore, the fourth dominant ST was ST26, which is frequently associated with clinical strains (Carroll et al., 2019). ST26 could be detected in all three types of aquatic products [Finfish (*n* = 5), mollusks (*n* = 2), and crustaceans (*n* = 3)]. Based on the cluster analysis, all isolates were grouped into 162 singletons and eight different clonal complexes, which included ST-142 complex (*n* = 48, 17.45%), ST-205 complex (*n* = 32, 11.64%), ST-18 complex (*n* = 20, 7.27%), ST-111 complex (*n* = 6, 2.18%), ST-365 complex (*n* = 3, 1.09%), ST-97 complex (n = 2, 0.73%), ST-8 complex (*n* = 1, 0.36%), and ST-23 complex (*n* = 1, 0.36%), indicating the overall high diversity of *B. cereus* from aquatic products.

Moreover, all isolates were divided into 13 clusters based on the MLST alleles with the threshold value of 52% similarity (Supplementary Figure S3). Cluster six contained most of the strains (74 strains) and cluster two only contained the strain Y273. Cluster seven had the highest proportion of strains displaying less antibiotic resistance and fewer virulence genes. In cluster seven, eight strains (26.7%) were resistant to less than six classes of antibiotics and 16 strains (53.3%) harbored fewer than six virulence genes. Emetic strains were not evenly distributed into different clusters, with only one strain each in clusters three, four, nine, and ten, two strains in cluster five and 10 strains in cluster six (eight *cesB*-positive isolates from aquatic products and two clinical emetic-type strains).

## Discussion

*B. cereus*, one of the most important foodborne pathogenic bacteria in China (Paudyal et al., 2018), causes different levels of food poisoning incidents (Schoeni and Wong, 2005; Logan, 2012). Our study here first examined the prevalence of *B. cereus* in retail aquatic products collected from 39 major cities of China. According to previous studies, the contamination rate of *B. cereus* in different kinds of food was from 6.8 to 57% (Iurlina et al., 2006; Flores-Urbán et al., 2014; Merzougui et al., 2014; Chon et al., 2015; Tewari et al., 2015; Zhang et al., 2017). In comparison, the contamination rate of *B. cereus* in our study was 25.47%, which is much higher than the rate in other reports, such as the one in Thailand aquatic products (5%; Ananchaipattana et al., 2012) or in American retail seafood (17.9%; Rahmati and Labbé, 2008). Since aquatic products can be rapidly oxidized and decomposed by microorganisms due to their high moisture content and high unsaturated fatty acid content, they are suitable habitats for *B. cereus* propagation. Besides, *B*. *cereus* can produce endospores that are resistant to heat stress (Stenfors Arnesen et al., 2008), particularly for short heat treatments for aquatic products. Thereby, *B. cereus* will be present in the cooked food where it may multiply and cause foodborne diseases. Considering the regional distribution, the contamination rate in 39 major cities of China ranged from zero (Hong Kong) to 50.00% (Shaoguan) (Supplementary Table S4). In terms of sample type, the highest proportion of food poisoning incidences caused by *B. cereus* was from crustaceans which was noted in the United States from 1973 to 2006 (Iwamoto et al., 2010). Of note, crustaceans collected in our study also had the highest contamination rate (27.78%), which reminds the consumers to pay attention to the way they consume crustaceans.

99.6% and 61.8% of the isolates contained *nheABC* cluster and *hblACD* group, respectively, which are slightly higher than those in retail seafood of United States (94% for *nheABC* and 50% for *hblACD*; Rahmati and Labbé, 2008). Moreover, the prevalence of the *nheABC* cluster in our isolates is higher than that in food products of Poland (78.6% for *nheABC*; Berthold-Pluta et al., 2019). *cytK*-*2* was detected in 93.1% of the isolates (256/275), which is much higher than the rate of previous reports (37.4–73%; Gao et al., 2018; Fiedler et al., 2019; Yu et al., 2019). Moreover, the proportion (>5%; 14/275) of the emetic strain is higher than the general level around the world (Chaabouni et al., 2015; Fiedler et al., 2019). The *cesB*-positive strains we isolated had both psychrotrophic and mesophilic signatures (Table 2 and Supplementary Figure S4), corresponding to an intermediate genotype according to von Stetten et al., 1998. In accordance with the previous study, most emetic strains are mesophilic and none of them have *hbl* genes, whereas a few emetic strains are psychrotrophic and may have *hbl* genes (Thorsen et al., 2006). In contrast, all *cesB*-positive isolates we identified were mesophilic as they were unable to grow at 7°C and possessed *hbl* genes (Table 2 and Supplementary Figure S4), which is different from previous reports (Ehling-Schulz et al., 2005; Thorsen et al., 2006). When considering the regional distribution, 12 of the *cesB*-positive isolates were identified from the samples collected from central, eastern, and southwest China. The climate in these areas is mostly the subtropical monsoon climate. Therefore, the geographical distribution of these mesophilic *cesB*-positive isolates may be in line with the regional climate.

According to the report of Ling et al. (2018), we also divided China into seven regions (Supplementary Figure S1). Of the 147 STs, 11 STs (7.48%) were detected in three or more regions. Among them, ST205 was detected in all regions except for the northern part of China. ST26, the common ST with clinical isolates (Carroll et al., 2019), was detected in four regions of China. 275 isolates from aquatic products and eight reference strains were classified into 13 clusters. Either cluster two or 13 has only one strain (Supplementary Figure S3). Most of the strains in clusters one, three, four, and nine were singleton and the profiles of virulence genes within the same cluster were quite similar (Supplementary Figure S3); however, the profiles of antimicrobial resistance were much diverse. In cluster seven, eight strains were resistant to less than six classes of antibiotics, most of which are common β-lactams, and none of these strains contained *hblA* virulence genes. The *cesB*-positive strains from aquatic products were mainly distributed in cluster six (8/14; 57.1%), which also contained two clinical emetic strains *B. cereus* F4810/72 and NC7401. Therefore, the pathogenic potential of these potential emetic strains within the cluster six should not be neglected. The other six *cesB*-positive strains were randomly distributed into different clusters, indicating that they may evolve from different origins. Additionally, these 14 strains possessed only two profiles of virulence genes [*nheA-nheB-nheC-hblC-hblD-cytK*-2*-cesB* (42.9%) or *nheA-nheB-nheC-hblA-hblC-hblD-cytK*-2*-cesB* (57.1%)] and the strains without *hblA* were only from ST26 and ST205 (Supplementary Figure S3).

*B. cereus* has been considered as an important bacterial pathogen through foodborne transmission (Glasset et al., 2016; Van Cauteren et al., 2017). For example, *B. cereus*, detected in both salmon (Labbé and Rahmati, 2012) and tuna (Doménech-Sánchez et al., 2011), produced either enterotoxin or vomiting toxin, respectively. There are some fatal cases through foodborne transmission by *B. cereus* (Veysseyre et al., 2015). Therefore, the application of effective antibiotics is very important for the clinical treatment of *B. cereus* if the case is very severe. The results of the antimicrobial analysis revealed that the most common resistant profile was AMP-P-AMC-KF-FOX-RD, which is as same as the profile of our previous study (Gao et al., 2018). Consistent with many reports (Luna et al., 2007; Owusu-Kwarteng et al., 2017), our results demonstrated that *B. cereus* has developed general resistance to β-lactam antibiotics. According to previous studies, *B. cereus* isolates also present resistance to different antibiotics used clinically, such as erythromycin, ciprofloxacin, clindamycin, chloramphenicol, etc. (Drobniewski, 1993; Tuladhar et al., 2000). In particular, the proportion of multidrug-resistant strains (99.64%) was higher than in previous reports (Gao et al., 2018; Yu et al., 2019), which may be due to the long-term use of different antibiotics in aquaculture (Alderman and Hastings, 1998; Martinez, 2009; Rico et al., 2013). According to the report by Wang et al. (2017), common medical antibiotics such as macrolides, tetracyclines, and fluoroquinolones have also been detected in aquatic products. It is worth noting that our *B. cereus* isolates in aquatic samples showed a high proportion of intermediate resistance (Figure 2). The overall situation reminds us to pay attention to the antibiotics used in aquaculture and potential hazard might be caused by these different virulent strains with serious antibiotic resistance.

With the increasing popularity of aquatic products in Chinese households and the favorable non-heated way to eat aquatic products, consumers and administrative departments should pay more attention to the potential pathogenic risk of *B. cereus.*

## Conclusion

Overall, 275 *B. cereus* isolates were identified in 219 aquatic products. Of all the samples, 1.83% (4/219) possessed >1,100 MPN/g *B. cereus* counting. Different isolates had virulence potential, among which 59.6% contained all three types of enterotoxin genes and 5.1% possessed *cesB.* Based on the MLST analysis, quite high genetic diversity was discovered, and the distribution of ST205 and ST26 in China was widespread. Given that *B. cereus* from aquatic products had a high proportion of intermediate resistance to different drugs, it is necessary to continuously monitor the antibiotic resistance of *B. cereus* in aquatic products and control antibiotics use in aquaculture.

## Data Availability Statement

All datasets generated for this study are included in the article/Supplementary Material.

## Author Contributions

YD, QW, JW, JumZ, YZ, and MeC conceived the project and designed the experiments. YZ, MeC, PY, SY, HG, JunZ, HuZ, MoC, HaZ, SW, RP, QY, LX, SZ, and YL performed the experiments. QW and YD supervised the project. YZ, MeC, and YD analyzed the data and wrote the manuscript. QW, JW, JumZ, and YD complemented the writing. All authors contributed to the article and approved the submitted version.

## Conflict of Interest

The authors declare that the research was conducted in the absence of any commercial or financial relationships that could be construed as a potential conflict of interest.

## Footnotes

1 http://pubmlst.org/bcereus/
