# Supplementary figures and images for: Prevalence, Virulence Feature, Antibiotic Resistance and MLST Typing of Bacillus cereus Isolated From Retail Aquatic Products in China
Source: Front Microbiol. 2020 Jul 3;11:1513. doi: 10.3389/fmicb.2020.01513 (PMC7347965; doi:10.3389/fmicb.2020.01513)

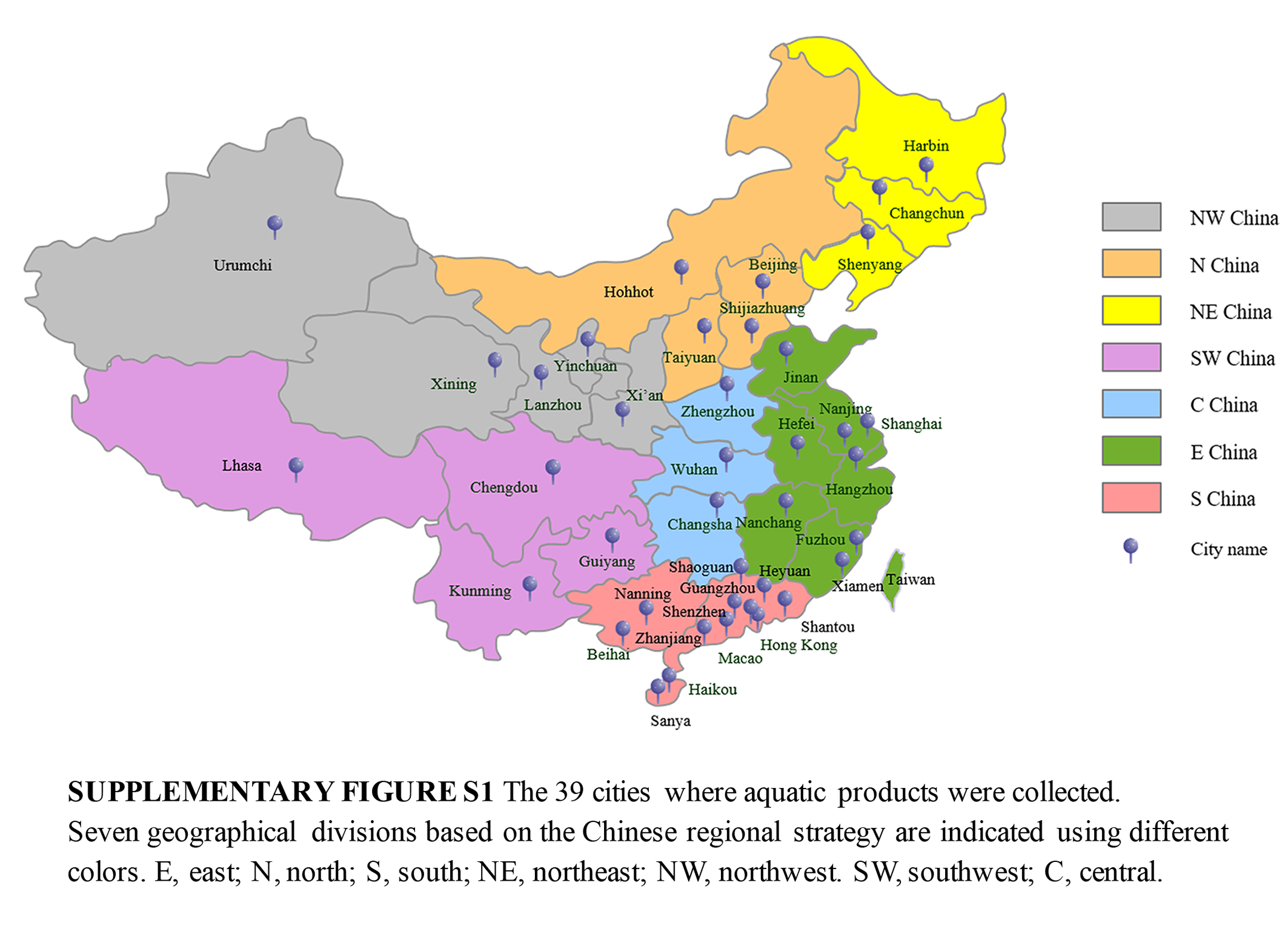

Supplement: Supplementary file 1 [file Image_1.TIF]

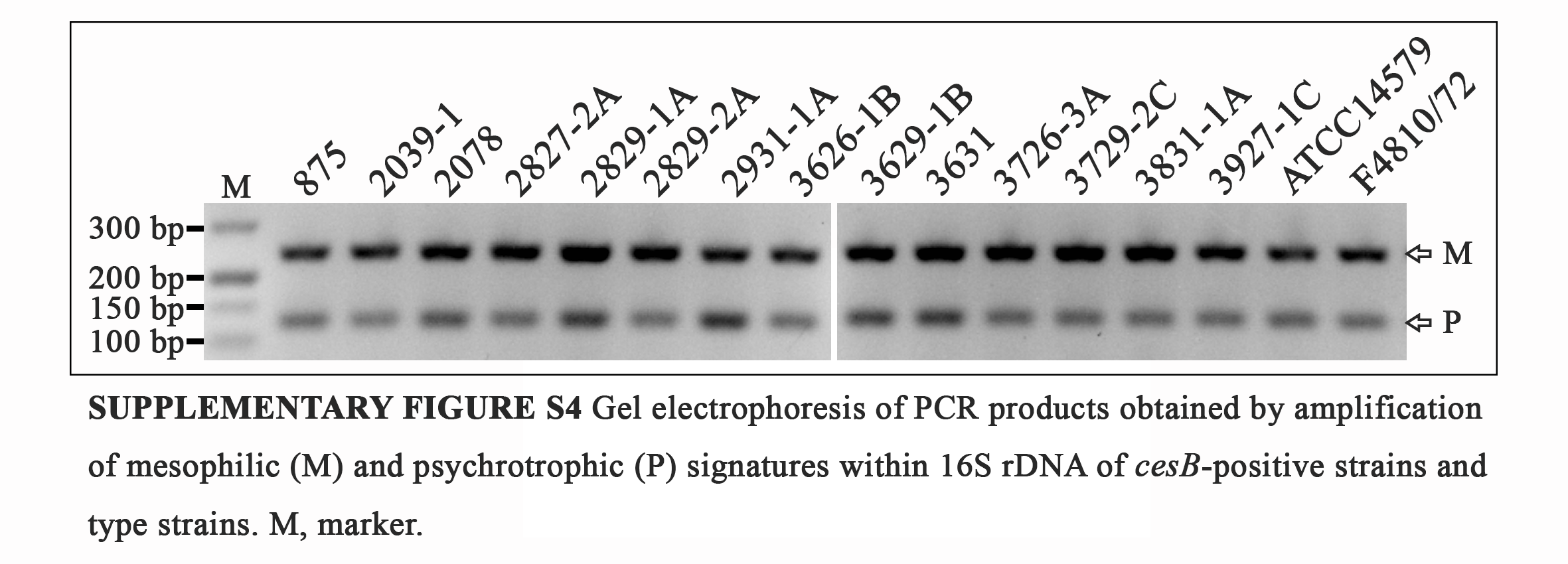

Supplement: Supplementary file 3 [file Image_3.TIF]
