# Supplementary material for: Prevalence, Virulence Feature, Antibiotic Resistance and MLST Typing of Bacillus cereus Isolated From Retail Aquatic Products in China
Source: Front Microbiol. 2020 Jul 3;11:1513. doi: 10.3389/fmicb.2020.01513 (PMC7347965; doi:10.3389/fmicb.2020.01513)
Supplement: Supplementary file 4 [file Data_Sheet_1.PDF]

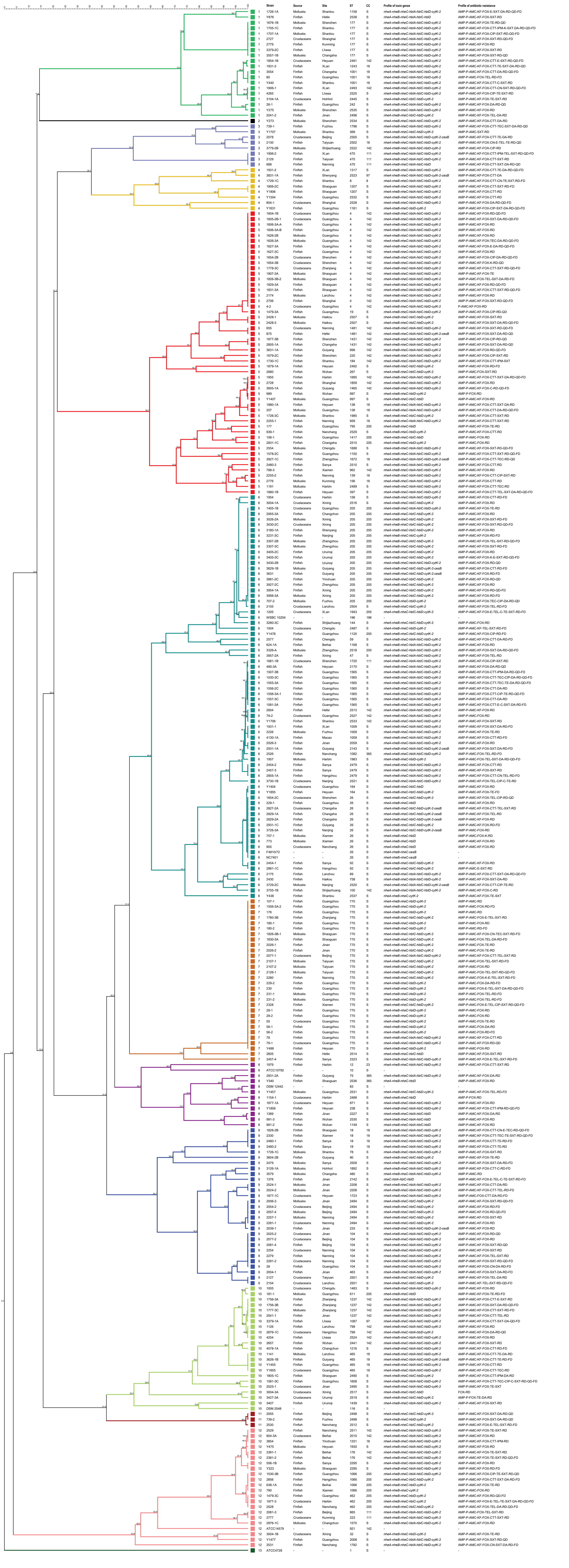

SUPPLEMENTARY FIGURE S3 Phylogenetic analysis of 275 *B. cereus* isolates from aquatic products and eight type strains based on MLST alleles.

A threshold value of 52% similarity was used to define different clusters. ATCC 14579: *B. cereus* ATCC 14579; DSM 2048: *Bacillus mycoides* DSM 2048; DSM 12442: *Bacillus pseudomycoides* DSM 12442; WSBC 10204: *Bacillus weihenstephanensis* WSBC 10204; ATCC 4728: *Bacillus anthracis* ATCC 4728; ATCC 10792: *Bacillus thuringiensis* ATCC 10792; NC7401: clinical emetic-type strain *B. cereus* NC7401; F4810/72: clinical emetic-type strain *B. cereus* F4810/72.
