# Supplementary material for: Prevalence, Virulence Feature, Antibiotic Resistance and MLST Typing of Bacillus cereus Isolated From Retail Aquatic Products in China
Source: Front Microbiol. 2020 Jul 3;11:1513. doi: 10.3389/fmicb.2020.01513 (PMC7347965; doi:10.3389/fmicb.2020.01513)
Supplement: Supplementary file 5 [file Table_1.DOCX]

**SUPPLEMENTARY TABLE S1** The most probable number (MPN) of *B. cereus* per gram (mL) samples.

| Number of positive tubes ^a^ | | | MPN/g | 95% confidence intervals | | Number of positive tubes | | | MPN  /g | 95% confidence intervals | |
| --- | --- | --- | --- | --- | --- | --- | --- | --- | --- | --- | --- |
| 0.10 | 0.01 | 0.001 |  | Low | High | 0.10 | 0.01 | 0.001 |  | Low | High |
| 0 | 0 | 0 | <3.0 | - | 9.5 | 2 | 2 | 0 | 21 | 4.5 | 42 |
| 0 | 0 | 1 | 3.0 | 0.15 | 9.6 | 2 | 2 | 1 | 28 | 8.7 | 94 |
| 0 | 1 | 0 | 3.0 | 0.15 | 11 | 2 | 2 | 2 | 35 | 8.7 | 94 |
| 0 | 1 | 1 | 6.1 | 1.2 | 18 | 2 | 3 | 0 | 29 | 8.7 | 94 |
| 0 | 2 | 0 | 6.2 | 1.2 | 18 | 2 | 3 | 1 | 36 | 8.7 | 94 |
| 0 | 3 | 0 | 9.4 | 3.6 | 38 | 3 | 0 | 0 | 23 | 4.6 | 94 |
| 1 | 0 | 0 | 3.6 | 0.17 | 18 | 3 | 0 | 1 | 38 | 8.7 | 110 |
| 1 | 0 | 1 | 7.2 | 1.3 | 18 | 3 | 0 | 2 | 64 | 17 | 180 |
| 1 | 0 | 2 | 11 | 3.6 | 38 | 3 | 1 | 0 | 43 | 9 | 180 |
| 1 | 1 | 0 | 7.4 | 1.3 | 20 | 3 | 1 | 1 | 75 | 17 | 200 |
| 1 | 1 | 1 | 11 | 3.6 | 38 | 3 | 1 | 2 | 120 | 37 | 420 |
| 1 | 2 | 0 | 11 | 3.6 | 42 | 3 | 1 | 3 | 160 | 40 | 420 |
| 1 | 2 | 1 | 15 | 4.5 | 42 | 3 | 2 | 0 | 93 | 18 | 420 |
| 1 | 3 | 0 | 16 | 4.5 | 42 | 3 | 2 | 1 | 150 | 37 | 420 |
| 2 | 0 | 0 | 9.2 | 1.4 | 38 | 3 | 2 | 2 | 210 | 40 | 430 |
| 2 | 0 | 1 | 14 | 3.6 | 42 | 3 | 2 | 3 | 290 | 90 | 1000 |
| 2 | 0 | 2 | 20 | 4.5 | 42 | 3 | 3 | 0 | 240 | 42 | 1000 |
| 2 | 1 | 0 | 15 | 3.7 | 42 | 3 | 3 | 1 | 460 | 90 | 2000 |
| 2 | 1 | 1 | 20 | 4.5 | 42 | 3 | 3 | 2 | 1100 | 180 | 4100 |
| 2 | 1 | 2 | 27 | 8.7 | 94 | 3 | 3 | 3 | >1100 | 420 | - |

1. This table uses 3 dilutions (0.1 g (mL), 0.01 g (mL) and 0.001 g (mL)) and 3 tubes per dilution.
