# Supplementary material for: Prevalence, Virulence Feature, Antibiotic Resistance and MLST Typing of Bacillus cereus Isolated From Retail Aquatic Products in China
Source: Front Microbiol. 2020 Jul 3;11:1513. doi: 10.3389/fmicb.2020.01513 (PMC7347965; doi:10.3389/fmicb.2020.01513)
Supplement: Supplementary file 6 [file Table_2.docx]

**SUPPLEMENTARY TABLE S2** Primers used in this study.

| **Primer** | **Sequence (5'-3')** | **Target fragment length (bp)** | **Annealing temperature (°C)** | **Reference** |
| --- | --- | --- | --- | --- |
| hblA-F | GTGCAGATGTTGATGCCGAT | 320 | 55 | (Hansen and Hendriksen, 2001) |
| hblA-R | ATGCCACTGCGTGGACATAT |  |  |  |
| hblC-F | AATGGTCATCGGAACTCTAT | 750 | 55 |  |
| hblC-R | CTCGCTGTTCTGCTGTTAAT |  |  |  |
| hblD-F | AATCAAGAGCTGTCACGAAT | 430 | 55 |  |
| hblD-R | CACCAATTGACCATGCTAAT |  |  |  |
| nheA-F | TACGCTAAGGAGGGGCA | 500 | 55 |  |
| nheA-R | GTTTTTATTGCTTCATCGGCT |  |  |  |
| nheB-F | CTATCAGCACTTATGGCAG | 770 | 55 |  |
| nheB-R | ACTCCTAGCGGTGTTCC |  |  |  |
| nheC-F | CGGTAGTGATTGCTGGG | 583 | 55 |  |
| nheC-R | CAGCATTCGTACTTGCCAA |  |  |  |
| cytK-2-F | AAAATGTTTAGCATTATCCGCTGT | 238 | 55 | (Oltuszak-Walczak and Walczak, 2013) |
| cytK-2-R | ACCAGTTGTATTAATAACGGCAATC |  |  |  |
| cesB-F | GGTGACACATTATCATATAAGGTG | 1271 | 58 | (Ehling-Schulz et al., 2005) |
| cesB-R | GTAAGCGAACCTGTCTGTAACAACA |  |  |  |
| glpF-F | GCGTTTGTGCTGGTGTAAGT | 549 | 59 | PubMLST (http://pubmlst.org/bcereus/ info/primers.shtml) |
| glpF-R | CTGCAATCGGAAGGAAGAAG |  |  |  |
| gmk-F | TTAAGTGAGGAAGGGTAGG | 600 | 56 |  |
| gmk-R | AATGTTCACCAACCACAA |  |  |  |
| ilvD-F | GGGCAAACATTAAGAGAA | 556 | 58 |  |
| ilvD-R | TTCTGGTCGTTTCCATTC |  |  |  |
| pta-F | AGAGCGTTTAGCAAAAGAA | 576 | 56 |  |
| pta-R | CAATGCGAGTTGCTTCTA |  |  |  |
| pur-F | GCTGCGAAAAATCACAAA | 536 | 56 |  |
| pur-R | CACGATTCGCTGCAATAA |  |  |  |
| pycA-F | GTTAGGTGGAAACGAAAG | 550 | 57 |  |
| pycA-R | CGTCCAAGTTTATGGAAT |  |  |  |
| tpi-F | CCAGTAGCACTTAGCGAC | 553 | 58 |  |
| tpi-R | GAAACCGTCAAGAATGAT |  |  |  |
| ERIC-F | ATGTAAGCTCCTGGGGATTCAC | 200 up | 45 | (Versalovic et al., 1991) |
| ERIC-R | AAGTAAGTGACTGGGGTGAGCG |  |  |  |
| MF | ATAACATTTTGAACCGCATG | 249 | 55 | (Stenfors and Granum, 2001; von Stetten et al., 1998) |
| UR | CTTCATCACTCACGCGGC |  |  |  |
| UF | CAAGGCTGAAACTCAAAGGA | 132 | 55 | (Stenfors and Granum, 2001; von Stetten et al., 1998) |
| PR | GAGAAGCTCTATCTCTAGA |  |  |  |
