# Supplementary material for: Prevalence, Virulence Feature, Antibiotic Resistance and MLST Typing of Bacillus cereus Isolated From Retail Aquatic Products in China
Source: Front Microbiol. 2020 Jul 3;11:1513. doi: 10.3389/fmicb.2020.01513 (PMC7347965; doi:10.3389/fmicb.2020.01513)
Supplement: Supplementary file 7 [file Table_3.docx]

**SUPPLEMENTARY TABLE S3** Twenty antibiotics used in antimicrobial susceptibility.

| Category | Antimicrobial class | Antimicrobial agent | Abbreviation | Zone Diameter Interpretive Criteria  (nearest whole mm) | | |
| --- | --- | --- | --- | --- | --- | --- |
|  |  |  |  | **R (≤)** | **I** | **S (≥)** |
| β-lactams | Penicillins | Ampicillin (10 μg) | AMP | 28 | - | 29 |
|  |  | Penicillin (10 units) | P | 28 | - | 29 |
|  | β-Lactam/β-lactamase inhibitor combinations | Amoxicillin-clavulanic acid  (20 μg / 10 μg) | AMC | 19 | - | 20 |
|  | Cephems (parenteral) | Cephalothin (30 μg) | KF | 14 | 15-17 | 18 |
|  |  | Cefoxitin (30 μg) | FOX | 21 | - | 22 |
|  |  | Cefotetan (30 μg) | CTT | 12 | 13-15 | 16 |
|  | Penems | Imipenem (10 μg) | IPM | 13 | 14-15 | 16 |
| Non-β-lactams | Aminoglycosides | Gentamicin (10 μg) | CN | 12 | 13-14 | 15 |
|  |  | Kanamycin (30 μg) | K | 13 | 14-17 | 18 |
|  | Macrolides | Erythromycin (15 μg) | E | 13 | 14-22 | 23 |
|  | Ketolide | Telithromycin (15 μg) | TEL | 18 | 19-21 | 22 |
|  | Glycopeptides | Teicoplanin (30 μg) | TEC | 10 | 11-13 | 14 |
|  | Quinolones | Ciprofloxacin (5 μg) | CIP | 15 | 16-20 | 21 |
|  | Phenylpropanol | Chloramphenicol (30 μg) | C | 12 | 13-17 | 18 |
|  | Tetracyclines | Tetracycline (30 μg) | TE | 14 | 15-18 | 19 |
|  | Folate pathway inhibitors | Trimethoprim-Sulfamethoxazole (1.25 μg / 23.75 μg) | SXT | 10 | 11-15 | 16 |
|  | Lincosamides | Clindamycin (2 μg) | DA | 14 | 15-20 | 21 |
|  | Ansamycins | Rifampin (5 μg) | RD | 16 | 17-19 | 20 |
|  | Streptogramins | Quinupristin-dalfopristin (15 μg) | QD | 15 | 16-18 | 19 |
|  | Nitrofurans | Nitrofurantoin (300 μg) | FD | 14 | 15-16 | 17 |

According to zone diameter interpretive criteria in the antibiotic resistance experiment, R, I and S represent the criteria of resistant, intermediate resistant, and sensitive isolates to different antibiotics, respectively (The Clinical and Laboratory Standards Institute [CLSI], 2010).
