# Supplementary material for: Prevalence, Virulence Feature, Antibiotic Resistance and MLST Typing of Bacillus cereus Isolated From Retail Aquatic Products in China
Source: Front Microbiol. 2020 Jul 3;11:1513. doi: 10.3389/fmicb.2020.01513 (PMC7347965; doi:10.3389/fmicb.2020.01513)
Supplement: Supplementary file 8 [file Table_4.DOCX]

**SUPPLEMENTARY TABLE S4** Prevalence of *B. cereus* isolated from aquatic samples in seven geographic regions and 39 cities of China.

| **China geographical division** | **Prevalence rate of geographical division (%)** | **City** | **Prevalence rate of city (%)** |
| --- | --- | --- | --- |
| **C** | 23.33 | Zhengzhou | 15.00 |
|  |  | Wuhan | 20.00 |
|  |  | Changsha | 35.00 |
| **E** | 25.63 | Fuzhou | 15.00 |
|  |  | Hangzhou | 20.00 |
|  |  | Hefei | 20.00 |
|  |  | Nanjing | 20.00 |
|  |  | Shanghai | 20.00 |
|  |  | Xiamen | 30.00 |
|  |  | Nanchang | 35.00 |
|  |  | Jinan | 45.00 |
| **N** | 20.00 | Hohhot | 10.00 |
|  |  | Shijiazhuang | 15.00 |
|  |  | Taiyuan | 25.00 |
|  |  | Beijing | 30.00 |
| **NE** | 20.00 | Shenyang | 10.00 |
|  |  | Changchun | 15.00 |
|  |  | Harbin | 35.00 |
| **NW** | 21.00 | Yinchuan | 10.00 |
|  |  | Xi’an | 15.00 |
|  |  | Urumqi | 20.00 |
|  |  | Xining | 30.00 |
|  |  | Lanzhou | 30.00 |
| **S** | 30.94 | Hong Kong | 0 |
|  |  | Macao | 5.00 |
|  |  | Haikou | 10.00 |
|  |  | Beihai | 20.00 |
|  |  | Zhanjiang | 20.00 |
|  |  | Sanya | 25.00 |
|  |  | Shenzhen | 35.00 |
|  |  | Nanning | 40.00 |
|  |  | Shantou | 40.00 |
|  |  | Guangzhou | 41.00 |
|  |  | Heyuan | 45.00 |
|  |  | Shaoguan | 50.00 |
| **SW** | 20.00 | Kunming | 15.00 |
|  |  | Chengdu | 20.00 |
|  |  | Lhasa | 15.00 |
|  |  | Guiyang | 30.00 |

C, central China; E, east China; N, north China; NE, northeast China; NW, northwest China; S, south China; SW, southwest China.
