# Supplementary material for: Prevalence, Virulence Feature, Antibiotic Resistance and MLST Typing of Bacillus cereus Isolated From Retail Aquatic Products in China
Source: Front Microbiol. 2020 Jul 3;11:1513. doi: 10.3389/fmicb.2020.01513 (PMC7347965; doi:10.3389/fmicb.2020.01513)
Supplement: Supplementary file 9 [file Table_5.DOCX]

**SUPPLEMENTARY TABLE S5** Information about genetic diversity of *B. cereus* isolated from aquatic products in China.

| **No.** | **Strain name** | **ST type** | **Clonal complex** | **No.** | **Strain name** | **ST type** | **Clonal complex** | |
| --- | --- | --- | --- | --- | --- | --- | --- | --- |
| 1 | 1604-1B | 4 | ST-142 complex | 17 | 2706 | 4 | ST-142 complex | |
| 2 | 1605-2B-1 | 4 | ST-142 complex | 18 | 4-2 | 4 | ST-142 complex | |
| 3 | 1606-3A-A | 4 | ST-142 complex | 19 | 1729-1C | 8 | ST-8 complex | |
| 4 | 1606-3A-B | 4 | ST-142 complex | 20 | 1979 | 12 | ST-23 complex | |
| 5 | 1626-2B | 4 | ST-142 complex | 21 | 1828-2B | 18 | ST-18 complex | |
| 6 | 1626-3A | 4 | ST-142 complex | 22 | 2330 | 18 | ST-18 complex | |
| 7 | 1627-3A | 4 | ST-142 complex | 23 | 2480-1 | 18 | ST-18 complex | |
| 8 | 1627-3C | 4 | ST-142 complex | 24 | 2480-2 | 18 | ST-18 complex | |
| 9 | 1654-2B | 4 | ST-142 complex | 25 | 1479-3A | 19 | Singleton | |
| 10 | 1654-3B | 4 | ST-142 complex | 26 | 1654-2C | 26 | Singleton | |
| 11 | 1778-3C | 4 | ST-142 complex | 27 | 229-1 | 26 | Singleton | |
| 12 | 1807-3A | 4 | ST-142 complex | 28 | 2827-2A | 26 | Singleton | |
| 13 | 1826-3B-2 | 4 | ST-142 complex | 29 | 2829-1A | 26 | Singleton | |
| 14 | 1829-3A | 4 | ST-142 complex | 30 | 2829-2A | 26 | Singleton | |
| 15 | 1831-3A | 4 | ST-142 complex | 31 | 2931-1C | 26 | Singleton | |
| 16 | 2174 | 4 | ST-142 complex | 32 | 3726-3A | 26 | Singleton | |
| 33 | 757-1 | 26 | Singleton | 51 | 2281-2 | 104 | Singleton | |
| 34 | 773 | 26 | Singleton | 52 | 29 | 104 | Singleton | |
| 35 | 905 | 26 | Singleton | 53 | 1880-1A | 138 | ST-18 complex | |
| 36 | 3004-1B | 32 | Singleton | 54 | 207 | 138 | ST-18 complex | |
| 37 | 3957-2A | 47 | Singleton | 55 | 2255-2 | 139 | ST-18 complex | |
| 38 | 2577 | 59 | Singleton | 56 | 3280-3C | 144 | Singleton | |
| 39 | 2931-2A | 75 | ST-365 complex | 57 | 2776 | 156 | ST-18 complex | |
| 40 | 1726-1C | 78 | Singleton | 58 | 1954 | 158 | Singleton | |
| 41 | 2175 | 89 | Singleton | 59 | Y1404 | 164 | Singleton | |
| 42 | 3604-2B | 90 | Singleton | 60 | Y1855 | 164 | Singleton | |
| 43 | 2454-1 | 92 | Singleton | 61 | 2381-1 | 176 | ST-142 complex | |
| 44 | 2881-1C | 92 | Singleton | 62 | 2381-2 | 176 | ST-142 complex | |
| 45 | 3755-1B | 100 | ST-142 complex | 63 | 1676-1B | 177 | Singleton | |
| 46 | 2025-2 | 104 | Singleton | 64 | 1705-1C | 177 | Singleton | |
| 47 | 2077-2 | 104 | Singleton | 65 | 1707-1A | 177 | Singleton | |
| 48 | 2081-4 | 104 | Singleton | 66 | 2727 | 177 | Singleton | |
| 49 | 2254 | 104 | Singleton | 67 | 2779 | 177 | Singleton | |
| 50 | 2279 | 104 | Singleton | 68 | 3379-2C | 177 | Singleton | |
| 69 | 3557-1B | 177 | Singleton | 87 | 3956-3A | 205 | ST-205 complex | |
| 70 | 1730-1C | 184 | ST-142 complex | 88 | 707-2 | 205 | ST-205 complex | |
| 71 | 1405-1B | 205 | ST-205 complex | 89 | 1679-2C | 220 | ST-142 complex | |
| 72 | 2955-3A | 205 | ST-205 complex | 90 | 2777 | 223 | ST-111 complex | |
| 73 | 3026-2A | 205 | ST-205 complex | 91 | 2039-1 | 233 | Singleton | |
| 74 | 3030-2C | 205 | ST-205 complex | 92 | Y1856 | 238 | Singleton | |
| 75 | 3180-1A | 205 | ST-205 complex | 93 | 26-1 | 242 | Singleton | |
| 76 | 3231-3C | 205 | ST-205 complex | 94 | 2680 | 267 | Singleton | |
| 77 | 3307-2B | 205 | ST-205 complex | 95 | Y1707 | 369 | Singleton | |
| 78 | 3307-3C | 205 | ST-205 complex | 96 | 1880-1B | 397 | Singleton | |
| 79 | 3405-2C | 205 | ST-205 complex | 97 | 1479-3C | 462 | ST-205 complex | |
| 80 | 3405-3C | 205 | ST-205 complex | 98 | 1977-3 | 462 | ST-205 complex | |
| 81 | 3430-2B | 205 | ST-205 complex | 99 | 2528 | 462 | ST-205 complex | |
| 82 | 3629-1B | 205 | ST-205 complex | 100 | 2004-1 | 463 | Singleton | |
| 83 | 3631 | 205 | ST-205 complex | 101 | 1141 | 465 | ST-18 complex | |
| 84 | 3881-2C | 205 | ST-205 complex | 102 | 3626-1B | 465 | ST-18 complex | |
| 85 | 3927-2C | 205 | ST-205 complex | 103 | Y1455 | 465 | ST-18 complex | |
| 86 | 3954-1A | 205 | ST-205 complex | 104 | Y1605 | 465 | ST-18 complex | |
| 105 | 1906-2 | 470 | ST-111 complex | 123 | 2107-1 | 770 | Singleton |  |
| 106 | 2129 | 470 | ST-111 complex | 124 | 2107-2 | 770 | Singleton |  |
| 107 | 688 | 470 | ST-111 complex | 125 | 2126-1 | 770 | Singleton |  |
| 108 | 3579 | 480 | Singleton | 126 | 2280 | 770 | Singleton |  |
| 109 | 181-1 | 611 | ST-205 complex | 127 | 229-2 | 770 | Singleton |  |
| 110 | 2081-3 | 665 | ST-111 complex | 128 | 230 | 770 | Singleton |  |
| 111 | 2430 | 758 | Singleton | 129 | 231-1 | 770 | Singleton |  |
| 112 | 107-1 | 770 | Singleton | 130 | 231-2 | 770 | Singleton |  |
| 113 | 1556-3A-2 | 770 | Singleton | 131 | 2328 | 770 | Singleton |  |
| 114 | 176 | 770 | Singleton | 132 | 28-1 | 770 | Singleton |  |
| 115 | 1780-3B | 770 | Singleton | 133 | 28-2 | 770 | Singleton |  |
| 116 | 180-1 | 770 | Singleton | 134 | 55 | 770 | Singleton |  |
| 117 | 180-2 | 770 | Singleton | 135 | 56-1 | 770 | Singleton |  |
| 118 | 1826-3B-1 | 770 | Singleton | 136 | 56-2 | 770 | Singleton |  |
| 119 | 1830-3A | 770 | Singleton | 137 | 78 | 770 | Singleton |  |
| 120 | 2026-1 | 770 | Singleton | 138 | 79-1 | 770 | Singleton |  |
| 121 | 2026-2 | 770 | Singleton | 139 | Y488 | 770 | Singleton |  |
| 122 | 2077-1 | 770 | Singleton | 140 | 177 | 795 | ST-205 complex |  |
| 141 | 1126 | 799 | ST-142 complex | 159 | 2526 | 1082 | ST-365 complex |  |
| 142 | 2876-1C | 799 | ST-142 complex | 160 | 3379-1A | 1087 | ST-97 complex |  |
| 143 | 1877-1A | 871 | Singleton | 161 | Y1478 | 1120 | ST-205 complex |  |
| 144 | 2255-1 | 959 | ST-18 complex | 162 | 991-2 | 1149 | Singleton |  |
| 145 | 788-3 | 962 | ST-142 complex | 163 | 1578-2C | 1150 | Singleton |  |
| 146 | 989 | 997 | Singleton | 164 | 1726-1A | 1159 | Singleton |  |
| 147 | Y1407 | 997 | Singleton | 165 | Y1631 | 1161 | Singleton |  |
| 148 | 3631-1A | 999 | ST-142 complex | 166 | 624-1A | 1168 | Singleton |  |
| 149 | 3554 | 1001 | ST-18 complex | 167 | 1806-2C | 1207 | Singleton |  |
| 150 | 80 | 1001 | ST-18 complex | 168 | Y1806 | 1207 | Singleton |  |
| 151 | Y440 | 1001 | ST-18 complex | 169 | 4078-1A | 1216 | Singleton |  |
| 152 | 1931-1 | 1009 | Singleton | 170 | 1756-3A | 1237 | ST-142 complex |  |
| 153 | 2226 | 1009 | Singleton | 171 | 1756-3B | 1237 | ST-142 complex |  |
| 154 | 4130-1A | 1009 | Singleton | 172 | 1777-3C | 1237 | ST-142 complex |  |
| 155 | 1530-3B | 1066 | ST-205 complex | 173 | 2041-1 | 1237 | ST-142 complex |  |
| 156 | 2856 | 1066 | ST-205 complex | 174 | 1931-3 | 1243 | ST-18 complex |  |
| 157 | 638-1A | 1066 | ST-205 complex | 175 | 1931-2 | 1317 | Singleton |  |
| 158 | 790 | 1066 | ST-205 complex | 176 | 3854 | 1331 | ST-18 complex |  |
| 177 | 106-1 | 1417 | ST-205 complex | 195 | 1955 | 1685 | ST-142 complex |  |
| 178 | 1677-3B | 1431 | ST-142 complex | 196 | 2554 | 1688 | Singleton |  |
| 179 | 2805-1A | 1431 | ST-142 complex | 197 | 1681-1B | 1720 | ST-111 complex |  |
| 180 | 3407 | 1439 | Singleton | 198 | 1877-1C | 1723 | Singleton |  |
| 181 | 3605-1A | 1465 | ST-142 complex | 199 | 2531 | 1782 | Singleton |  |
| 182 | 655 | 1481 | ST-142 complex | 200 | 739-1 | 1796 | Singleton |  |
| 183 | 875 | 1481 | ST-142 complex | 201 | 2728 | 1859 | ST-142 complex |  |
| 184 | 1005 | 1483 | Singleton | 202 | 3126-1A | 1892 | Singleton |  |
| 185 | 1507-3B | 1565 | Singleton | 203 | Y475 | 1930 | Singleton |  |
| 186 | 1530-3C | 1565 | Singleton | 204 | 1205 | 1943 | ST-205 complex |  |
| 187 | 1555-3A | 1565 | Singleton | 205 | 1957 | 1983 | Singleton |  |
| 188 | 1556-2C | 1565 | Singleton | 206 | 1726-3C | 1985 | Singleton |  |
| 189 | 1556-3A-1 | 1565 | Singleton | 207 | Y1477 | 2008 | Singleton |  |
| 190 | 1557-3C | 1565 | Singleton | 208 | 604-3A | 2010 | ST-142 complex |  |
| 191 | 1581-3A | 1565 | Singleton | 209 | 2026-3 | 2059 | Singleton |  |
| 192 | 2976-1C | 1570 | Singleton | 210 | 1376 | 2142 | Singleton |  |
| 193 | 1581-3C | 1608 | Singleton | 211 | 2931-1A | 2143 | Singleton |  |
| 194 | 3927-1C | 1672 | ST-18 complex | 212 | 490-3A | 2170 | Singleton |  |
| 213 | 2024-1 | 2208 | Singleton | 231 | 2006-3 | 2494* | Singleton |  |
| 214 | 2024-2 | 2208 | Singleton | 232 | 2054-2 | 2494* | Singleton |  |
| 215 | 1389 | 2227 | Singleton | 233 | 2057-4 | 2494* | Singleton |  |
| 216 | 556-1B | 2295 | Singleton | 234 | 2257-1 | 2494* | Singleton |  |
| 217 | Y323 | 2295 | Singleton | 235 | 2281-1 | 2494* | Singleton |  |
| 218 | 2457-4 | 2323 | Singleton | 236 | 2025-1 | 2495* | Singleton |  |
| 219 | 2657 | 2441 | ST-142 complex | 237 | 2041-2 | 2496* | Singleton |  |
| 220 | 3104-1A | 2445 | Singleton | 238 | 2055 | 2498* | Singleton |  |
| 221 | 2454-2 | 2479 | Singleton | 239 | 739-2 | 2498* | Singleton |  |
| 222 | 2457-3 | 2479 | Singleton | 240 | 2078 | 2500* | Singleton |  |
| 223 | 2855-1A | 2479 | Singleton | 241 | 2127 | 2501* | Singleton |  |
| 224 | 1004 | 2487* | Singleton | 242 | 2154 | 2501* | Singleton |  |
| 225 | 1154-1 | 2488* | Singleton | 243 | 2130 | 2502* | ST-18 complex |  |
| 226 | 1191 | 2489* | Singleton | 244 | 2155 | 2504* | Singleton |  |
| 227 | 1805-1C | 2490* | Singleton | 245 | 2428-1 | 2507* | Singleton |  |
| 228 | 1854-1B | 2491* | ST-142 complex | 246 | 2428-3 | 2507* | Singleton |  |
| 229 | 1879-1A | 2492* | Singleton | 247 | 2479 | 2509* | Singleton |  |
| 230 | 1906-1 | 2493* | ST-142 complex | 248 | 2480-3 | 2510* | Singleton |  |
| 249 | 2529 | 2511* | ST-142 complex | 266 | 939-1 | 2529* | Singleton |  |
| 250 | 2530 | 2512* | Singleton | 267 | 991-3 | 2530* | Singleton |  |
| 251 | 2604 | 2513* | ST-142 complex | 268 | Y1457 | 2531* | Singleton |  |
| 252 | 2605 | 2514* | Singleton | 269 | Y1554 | 2532* | Singleton |  |
| 253 | 2831-1C | 2515* | ST-205 complex | 270 | Y1706 | 2533* | ST-142 complex |  |
| 254 | 3004-1A | 2516* | Singleton | 271 | Y273 | 2534* | Singleton |  |
| 255 | 3004-3A | 2517* | Singleton | 272 | Y275 | 2535* | Singleton |  |
| 256 | 3326-A | 2518* | ST-205 complex | 273 | Y340 | 2536* | ST-365 complex |  |
| 257 | 3427-3A | 2519* | Singleton | 274 | Y438 | 2537* | Singleton |  |
| 258 | 3729-2C | 2520* | Singleton | 275 | Y876 | 2538* | Singleton |  |
| 259 | 3730-1B | 2521* | Singleton |  |  |  |  |  |
| 260 | 3779-2B | 2522* | ST-142 complex |  |  |  |  |  |
| 261 | 3831-1A | 2523* | ST-97 complex |  |  |  |  |  |
| 262 | 4254 | 2524* | ST-142 complex |  |  |  |  |  |
| 263 | 4265 | 2525* | Singleton |  |  |  |  |  |
| 264 | 79-2 | 2527* | ST-142 complex |  |  |  |  |  |
| 265 | 804-1 | 2528* | Singleton |  |  |  |  |  |

* represents the new ST.
